# Hormonal responses to non-nutritive sweeteners in water and diet soda

**DOI:** 10.1186/s12986-016-0129-3

**Published:** 2016-10-21

**Authors:** Allison C. Sylvetsky, Rebecca J. Brown, Jenny E. Blau, Mary Walter, Kristina I. Rother

**Affiliations:** 1 Section on Pediatric Diabetes & Metabolism. DEOB, NIDDK, National Institutes of Health, 9000 Rockville Pike, Building 10, Room 8C432A, Bethesda, MD 20892 USA; 2 Department of Exercise and Nutrition Sciences, Milken Institute School of Public Health, The George Washington University, Washington, DC, USA; 3 Sumner M. Redstone Global Center for Prevention and Wellness, Milken Institute School of Public Health, The George Washington University, Washington, DC, USA; 4 Office of the Director, NIDDK, National Institutes of Health, Bethesda, MD USA

**Keywords:** Sucralose, Acesulfame-potassium, Diet soda, Non-nutritive sweetener, Gut peptides

## Abstract

**Background:**

Non-nutritive sweeteners (NNS), especially in form of diet soda, have been linked to metabolic derangements (e.g. obesity and diabetes) in epidemiologic studies. We aimed to test acute metabolic effects of NNS in isolation (water or seltzer) and in diet sodas.

**Methods:**

We conducted a four-period, cross-over study at the National Institutes of Health Clinical Center (Bethesda, Maryland). Thirty healthy adults consumed 355 mL water with 0 mg, 68 mg, 170 mg, and 250 mg sucralose, and 31 individuals consumed 355 mL caffeine-free Diet Rite Cola™, Diet Mountain Dew™ (18 mg sucralose, 18 mg acesulfame-potassium, 57 mg aspartame), and seltzer water with NNS (68 mg sucralose and 41 mg acesulfame-potassium, equivalent to Diet Rite Cola™) in randomized order, prior to oral glucose tolerance tests. Blood samples were collected serially for 130 min. Measures included GLP-1, GIP, glucose, insulin, C-peptide, glucose absorption, gastric emptying, and subjective hunger and satiety ratings.

**Results:**

Diet sodas augmented active GLP-1 (Diet Rite Cola™ vs. seltzer water, AUC, *p* = 0.039; Diet Mountain Dew™ vs. seltzer water, AUC, *p* = 0.07), but gastric emptying and satiety were unaffected. Insulin concentrations were nominally higher following all NNS conditions without altering glycemia. Sucralose alone (at any concentration) did not affect metabolic outcomes.

**Conclusions:**

Diet sodas but not NNS in water augmented GLP-1 responses to oral glucose. Whether the trends toward higher insulin concentrations after NNS are of clinical importance remains to be determined. Our findings emphasize the need to test metabolic effects of NNS after chronic consumption.

**Trial registration:**

The data for this manuscript were gathered from clinical trial #NCT01200940.

**Electronic supplementary material:**

The online version of this article (doi:10.1186/s12986-016-0129-3) contains supplementary material, which is available to authorized users.

## Background

Several large epidemiologic studies have suggested adverse metabolic effects resulting from non-nutritive sweeteners (NNS) consumption, surprisingly similar to sugar intake. These include weight gain [1], central adiposity [2], metabolic syndrome [3], and cardiovascular disease [4]. In two recent studies, consumption of regular and diet soft drinks was associated with a similarly increased risk of developing type 2 diabetes [5, 6] and non-alcoholic fatty liver disease [7], though associations were no longer significant among diet beverage consumers after adjustment for body weight. This finding suggests that obesity is the link between NNS and metabolic disease. In contrast to epidemiologic findings, randomized controlled trials with NNS have shown neutral effects or even possible weight management benefits following NNS consumption [8, 9]. This may be especially true when sugar is replaced with NNS [10]. An excellent overview of the existing controversies has recently been published [11].

While reverse causality likely contributes to the observed epidemiologic association between NNS use and weight [12], alternative explanations include behavioral and cognitive mechanisms such as greater energy intake after ‘saved’ calories due to choosing NNS [12] and a disruption of the predictive relationship between sweet taste perception and caloric intake [13]. Furthermore, NNS have been shown to induce less central reward compared to caloric sugars [14], potentially leading to continued seeking of palatable food. Recently, alterations of the gut microbiome have been reported after NNS exposure [15, 16], promoting greater energy harvest [17]. Based on in vitro studies, it has also been suggested that NNS may up-regulate adipogenesis [18]. It is important to note that NNS induced microbiome changes have only been reported in one small human study and changes in adipose tissue have not yet been replicated in humans.

Another plausible explanation is direct stimulation of insulin secretion in response to binding of NNS to sweet taste receptors (T1R2/T1R3) on pancreatic beta-cells. This has been documented in vitro [19, 20] and is supported by results of a small clinical trial showing increased insulin levels following NNS exposure in humans [21]. Sweet taste receptors are also found in the intestine [22], where they modulate various gut hormone responses, including glucagon-like-peptide 1 (GLP-1) secreted from enteroendocrine L-cells [23] and gastric inhibitory peptide (GIP) secreted from enteroendocrine K-cells [24]. We previously demonstrated that ingestion of a diet soda (Diet Rite Cola™) sweetened with sucralose and acesulfame-potassium, administered prior to an oral glucose load, resulted in a 34 % increase in GLP-1 secretion in comparison to unflavored carbonated water. This was shown both in healthy adolescents and young adults [25] and in youth with type 1 diabetes [26]. Herein we report the results of a randomized same-subject crossover study testing the effects of NNS on glycemia, insulin, and incretin responses in healthy adults. In the first set of experiments (study arm 1), we evaluated three doses of sucralose mixed in water to further test our original hypothesis that sucralose was the active ingredient in Diet Rite Cola™ responsible for the previously observed increase in glucose-dependent GLP-1 secretion. In the second set of experiments (study arm 2), we tested whether two combinations of NNS in diet sodas and in seltzer water increase GLP-1 secretion.

## Methods

Sixty-one healthy adults (30 participants in study arm 1 and 31 participants in arm 2) were enrolled. All subjects provided written informed consent. The protocol was approved by the Institutional Review Board of the National Institute of Diabetes and Digestive and Kidney Diseases (NIDDK) (NCT #01200940, Metabolic Effects of Non-nutritive Sweeteners). Inclusion criteria were age between 18 and 45 years and no known active medical conditions. Exclusion criteria included diabetes, pregnancy, lactation, and active medication use, other than oral contraceptives.

Study participants arrived at the NIH Clinical Center at approximately 8 am following a ten hour fast. They were instructed to avoid foods and beverages containing NNS, acetaminophen, and alcohol for two days prior to each study visit and to maintain a similar diet and physical activity level. Diet and physical activity during the 24 h prior to each study visit were recorded. Habitual NNS intake was assessed with a questionnaire and individuals were categorized based on the frequency of NNS use (whether in foods or beverages): at least once daily, less than daily but at least once per month, or never. In both study arms, visits were scheduled on four separate days, approximately one week apart to avoid carryover effects from the prior visit and to avoid any significant changes in body weight or metabolic parameters.

**Fig. 1:**
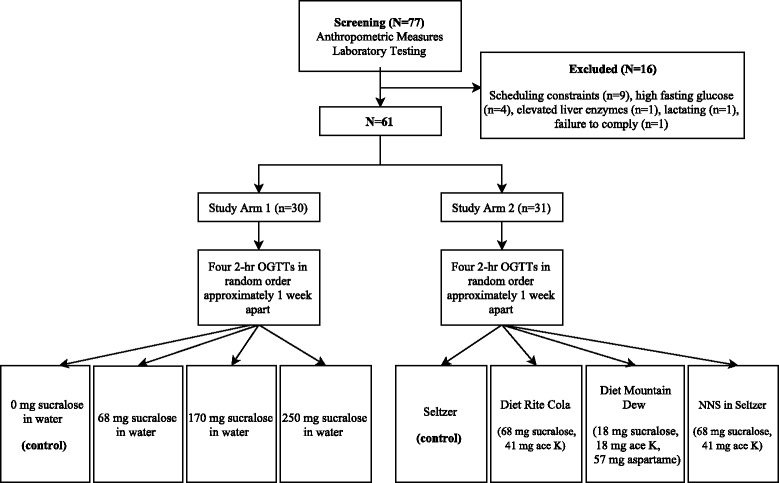
Following a screening visit, subjects were randomized to consume either 355 mL water mixed with a sucralose dose of 0 mg (plain water control), 68 mg, 170 mg, or 250 mg (study arm 1) or 355 mL seltzer water (control), 355 mL caffeine-free Diet Rite Cola™ sweetened with 68 mg sucralose and 41 mg acesulfame-potassium, 355 mL caffeine-free Diet Mountain Dew™ sweetened with 18 mg sucralose, 18 mg acesulfame-potassium and 57 mg aspartame or 68 mg sucralose and 41 mg acesulfame-potassium in 355 mL of seltzer water (study arm 2) prior to an OGTT

Block randomization (block size 24) based on a random number table was used to assign each subject to the four test beverage conditions. In study arm 1, subjects were randomized to consume 355 mL water mixed with a sucralose dose of 0 mg (plain water control), 68 mg, 170 mg, or 250 mg prior to an oral glucose tolerance test (OGTT). In study arm 2, subjects were randomized to consume 355 mL (standard 12 ounce can) seltzer water (control), 355 mL caffeine-free Diet Rite Cola™ sweetened with 68 mg sucralose and 41 mg acesulfame-potassium, 355 mL caffeine-free Diet Mountain Dew™ sweetened with 18 mg sucralose, 18 mg acesulfame-potassium and 57 mg aspartame, or 68 mg sucralose and 41 mg acesulfame-potassium in 355 mL of seltzer water prior to an OGTT. Study procedures are summarized in Fig. 1. Composition of the diet sodas are presented in Additional file 1: Table S1.

Baseline blood samples were drawn at −10 min, after which subjects consumed the assigned test beverage in 2–3 min. At time 0 min, a second blood sample was obtained, after which subjects ingested a standard oral glucose load (75 g glucose) mixed with 1450 mg acetaminophen and 7.5 g 3-*O*-methylglucose. Blood samples were drawn at 10, 20, 30, 60, 90, and 120 min following consumption of the glucose load. Satiety questionnaires were administered at baseline, immediately following consumption of the glucose load and after 30, 60, 90, and 120 min.

Study data were collected and managed using REDCap (Research Electronic Data Capture) hosted at Vanderbilt University.

### Measures

Active GLP-1 was measured by enzyme-linked immunosorbent assay (Millipore, Billerica MA, USA). The lowest detectable level of active GLP-1 was 6.56 pmol/L (inter-assay CV 8 % and intra-assay CV 7 %). In study arm 1, GIP was measured by enzyme-linked immunosorbent assay (Millipore, Billerica MA, USA). The lowest detectable level of GIP was 8.2 pg/mL inter-assay CV 1.8–6.1 % and intra-assay CV 3.0–8.8 %). In study arm 2, GIP was measured using the Milliplex gut hormone kit based on Luminex technology (EMD Millipore, Billerica MA, USA). The lowest detectable level for GIP was 0.2 pg/mL (inter-assay CV 7.0 % and intra-assay CV 5.1 %). DPP-IV inhibitor was added to blood collection tubes prior to sample collection for analysis of GLP-1 and GIP. In both study arms, insulin was measured using an ELISA (R&D Systems, Minneapolis, MN, intra-assay CV 1.9 % and inter-assay CV 4.1 %, assay range 15.6–500 pmol/L). Serum glucose was determined using the glucose oxidase method (inter-assay CV 3.9 % at 2.4 mmol/L and 1.2 % at 22.1 mmol/L; intra-assay CV 2.9 % at 2.4 mmol/L and 0.4 % at 22.1 mmol/L). C-peptide was measured by electrochemiluminescence immunoassay. Normal fasting range was 0.9–7.1 ng/mL in study arm 1 and 1.1–5.0 ng/mL in study arm 2. Glucose absorption was measured using 3-*O*-methylglucose (3-OMG, 7.5 g administered with the glucose load), which is an inert, non-metabolizable glucose analog. The appearance of 3-OMG in blood can thus be used as a proxy measure of the rate of intestinal glucose absorption [27]. Acetaminophen appearance in the blood was used as a proxy measure of the rate of gastric emptying [28]. 3-OMG and acetaminophen were analyzed by gas chromatography–mass spectrometry-(GC-MS) using a deuterated analyte as internal standard. Plasma was deproteinated, dried, and derivatized with methylboronic acid in pyridine for 3-OMG analysis. Acetaminophen was purified from acidified plasma by solid phase extraction, then silylated prior to analysis. All assays were performed in duplicate. Hunger and satiety were measured using 100 mm visual analog scales.

### Statistical analysis

The primary end-point in study arm 1 was the difference in glucose-stimulated active GLP-1 area-under-the-curve (AUC) following ingestion of 68 mg of sucralose and glucose-stimulated active GLP-1 secretion after the unsweetened water control. In study arm 2, our primary outcome was the difference between glucose-stimulated active GLP-1 AUC after ingestion of Diet Rite Cola™ and glucose-stimulated active GLP-following the unsweetened seltzer water control. Sample sizes were determined based on our prior study [25], where ingestion of Diet Rite Cola™ augmented glucose-stimulated GLP-1 response compared to an unsweetened seltzer water control. The difference in glucose-stimulated insulin secretion following ingestion of Diet Rite Cola™ compared to ingestion of seltzer water was evaluated as a secondary outcome.

**Fig. 2:**
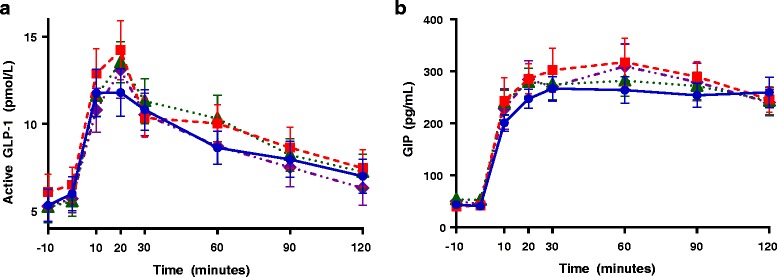
Serial data from OGTTs. Active glucagon-like-peptide 1 (GLP-1) (**a**) and gastric inhibitory peptide (GIP) (**b**) are shown after ingestion of either Diet Rite Cola™ ( with dashed line), Diet Mountain Dew™ ( with dotted line), 68 mg sucralose and 41 mg acesulfame-potassium in seltzer water ( with dashed and dotted line) or seltzer water ( with solid line) 10 min prior to a 75 g oral glucose load. Active GLP-1 was augmented in all three NNS conditions vs. the seltzer water condition. All data are expressed as mean ± standard error

**Fig. 3:**
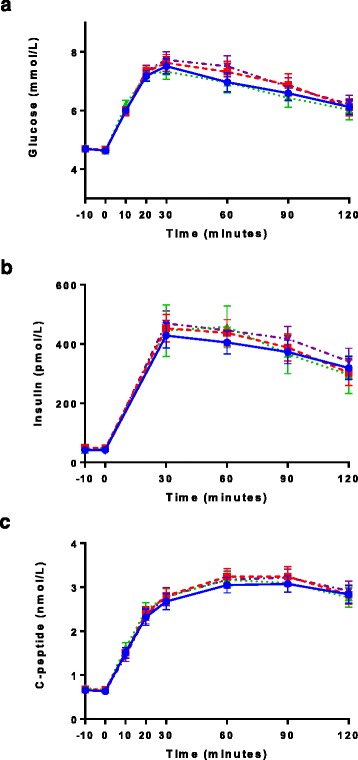
Serial data from OGTTs. Glucose (**a**), insulin (**b**), and C-peptide (**c**) are shown after ingestion of Diet Rite Cola™ ( with dashed line), Diet Mountain Dew™ ( with dotted line), 68 mg sucralose and 41 mg acesulfame-potassium ( with dashed and dotted line) or seltzer water ( with solid line). Insulin AUCs were 22 to 25% higher following all three NNS conditions vs. seltzer water (not statistically significant). All data are expressed as mean ± standard error

Descriptive statistics were calculated for each outcome of interest during each of the four test visits in both study arms. Incremental AUC was calculated using the trapezoidal method. Peak was calculated as the maximum value for each variable of interest over the 120-min time course and occurred at different time points (e.g. at 20 min or 30 min) for different individuals. Thus, peak values presented in Tables 2 and 3 differ slightly from those depicted in the response curves presented in Fig. 2 and Fig. 3. Differences between the mean peak and AUC in the four conditions were first assessed using repeated-measures ANOVA, and post-hoc Dunnett’s tests were used for pairwise comparisons, where repeated-measures ANOVA indicated a trend for difference between the groups. AUC values that were not normally distributed were log-transformed before analyses. No adjustments were made to account for multiple comparisons. Linear mixed modeling was used to account for fixed and random effects, given the same-subject crossover design of the study.

## Results

Characteristics of the study participants at baseline are shown in Table 1. Study participants were similar in age and gender, but more ethnically and racially diverse in study arm 2. Overall NNS consumption was low in both arms relative to the general population [29] with slightly higher consumption in study arm 2. The mean BMI was similar (25.8 ± 4.2 kg/m^2^ and 26.3 ± 7.5 kg/m^2^, respectively), but baseline insulin, C-peptide and HOMA were higher in study arm 2.

**Table 1:** Characteristics of healthy adult volunteers

Variable	Study arm 1 (mean ± SD)	Study arm 2 (mean ± SD)	*p*-value^b^
Age (years)	29.7 ± 7.6	27.4 ± 6.7	0.21
Female Gender	53 %	55 %	0.81
Race (%)			**0.02**
White	60 %	42 %	
Black	37 %	32 %	
Other	3 %	26 %	
Body Mass Index (BMI, kg/m^2^)	25.8 ± 4.2	26.3 ± 7.5	0.77
Weight Status (%)			0.56
Normal Weight	53 %	58 %	
Overweight	23 %	16 %	
Obese	23 %	26 %	
Glucose (umol/L)	4.6 ± 0.3	4.8 ± 0.35	0.08
Insulin (pmol/L)	32.7 ± 15.6	51.5 ± 42.1	**0.03**
C-Peptide (nmol/L)	0.4 ± 0.1	0.7 ± 0.3	**0.0006**
HOMA-2	0.9 ± 0.3	1.5 ± 0.7	**0.0001**
Hemoglobin A1c (%)	5.3 ± 0.3	5.2 ± 0.4	0.32
Triglycerides (mmol/L)	0.8 ± 0.3	1.1 ± 0.7	0.07
HDL (mmol/L)	1.5 ± 0.3	1.5 ± 0.4	0.87
LDL (mmol/L)	2.4 ± 0.7	2.4 ± 0.8	0.95
Total cholesterol (mmol/L)	4.3 ± 0.8	4.4 ± 0.9	0.61
NNS Beverage Consumption (%)^a^			**0.005**
Never	57 %	40 %	
≥ 1/month	26 %	53 %	
≥ 1/day	17 %	7 %	
NNS Packet Use (%)^a^			0.52
Never	70 %	73 %	
≥ 1/month	23 %	17 %	
≥ 1/day	7 %	10 %	

^a^Different questionnaires were used to assess NNS consumption in Study Arm 1 and Study Arm 2

^b^Values in bold reflect statistical significance using a cut-off of p<0.05

No statistically significant differences in active GLP-1, GIP, glucose, insulin, or C-peptide were observed when sucralose (at varying doses mixed in water) was administered before the OGTTs in study arm 1 (Table 2). In contrast, active GLP-1 AUC was higher following Diet Rite Cola™ (*p* = 0.04), and tended to be higher following Diet Mountain Dew™ (*p* = 0.07) (Fig. 2, Table 3), but not following the preload containing sucralose and acesulfame-potassium mixed in seltzer water. GIP results following Diet Rite Cola™ showed a similar trend, but did not reach statistical significance (peak *p* = 0.07). Glucose concentrations were indistinguishable between the four conditions (Fig. 3, Table 3). Both peak insulin and insulin AUC were 17 to 25 % higher after Diet Rite Cola™, Diet Mountain Dew™, and sucralose and acesulfame-potassium in seltzer water pre-treatments, but did not reach statistical significance. AUCs and peaks for active GLP-1, GIP, glucose, insulin, and C-peptide following Diet Rite Cola™, Diet Mountain Dew™, sucralose and acesulfame-potassium in seltzer water, and seltzer water pre-treatment are shown in Table 3.

**Table 2:** Glycemic and hormonal responses to an OGTT are similar after pretreatment with sucralose

Variable	0 mg sucralose (mean ± SD)	68 mg sucralose (mean ± SD)	170 mg sucralose (mean ± SD)	250 mg sucralose (mean ± SD)
Active GLP-1 AUC (pmol/L/120 min)	1268.4 ± 936.9	1217.7 ± 960.2	1327.4 ± 857.7	1328.4 ± 953.2
Active GLP-1 Peak (pmol/L)	18.2 ± 12.9	16.3 ± 11.1	17.4 ± 10.5	18.5 ± 14.4
GIP AUC (pg/mL/120 min)	13478.2 ± 6328.9	13298.0 ± 5877.8	13646.9 ± 6351.8	13881.6 ± 6720.2
GIP Peak (pg/mL)	139.9 ± 68.3	145.8 ± 80.2	141.0 ± 72.0	143.7 ± 68.2
Glucose AUC (mmol/L/120 min)	750.1 ± 149.2	766.6 ± 148.1	744.8 ± 134.6	730.8 ± 130.7
Glucose Peak (mmol/L)	7.1 ± 1.6	7.3 ± 1.5	7.1 ± 1.4	7.0 ± 1.3
Insulin AUC (pmol/L/120 min)	38571.6 ± 13814.6	39984.9 ± 24316.9	37749.1 ± 16896.9	36437.9 ± 13553.3
Insulin Peak (pmol/L)	420.1 ± 185.6	414.6 ± 182.5	404.1 ± 196.4	374.2 ± 147.8
C-Peptide AUC (nmol/L/120 min)	274.7 ± 71.8	278.3 ± 70.1	272.3 ± 84.1	269.7 ± 614.7
C-Peptide Peak (nmol/L)	3.0 ± 0.8	3.2 ± 0.8	3.1 ± 1.1	3.0 ± 0.8

**Table 3:** Glycemic and hormonal responses during oral glucose tolerance tests (OGTT) in study arm 2

Variable	Seltzer water (mean ± SD)	Diet Rite Cola™ (mean ± SD)	Diet Mountain Dew™ (mean ± SD)	Sucralose and Ace-K (mean ± SD)	*p*-value (ANOVA)
Active GLP-1 AUC (pmol/L/120 min)	1142.9 ± 702.8	1274.5 ± 781.8**	1268.5 ± 739.7	1173.2 ± 755.0	0.087
Active GLP-1 Peak (pmol/L)	14.3 ± 8.1	16.1 ± 9.9	14.5 ± 6.5	14.4 ± 8.6	0.53
Time to Active GLP-1 Peak (minutes)	24.2 ± 24.7	20.0 ± 13.6	23.0 ± 13.7	25.2 ± 22.9	0.73
GIP AUC (pg/mL/120 min)	30926.1 ± 14737.3	33652.6 ± 22175.9	31624.2 ± 16665.2	32132.3 ± 20268.2	0.62
GIP Peak (pg/mL)	335.8 ± 158.9	367.5 ± 260.1	329.6 ± 167.1	351.4 ± 266.5	0.43
Time to GIP Peak (minutes)	60.3 ± 35.5	55 ± 30.7	62.3 ± 40.1	55.5 ± 35.2	0.76
Glucose AUC (umol/L/120 min)	850.2 ± 142.8	873.3 ± 171.4	843.3 ± 170.5	880.6 ± 163.2	0.40
Glucose Peak (umol/L)	8.0 ± 1.4	8.4 ± 1.6	8.1 ± 1.7	8.4 ± 1.6	0.25
Time to Glucose Peak (minutes)	43.5 ± 28.8	44 ± 32.0	37.0 ± 24.4	41.3 ± 24.9	0.45
Insulin AUC (pmol/L/120 min)	55538.4 ± 28652.2	69164.8 ± 44218.6	67883.8 ± 41168.7	68039.7 ± 44373.7	0.53
Insulin Peak (pmol/mL)	734.7 ± 409.9	876.1 ± 490.8	862.4 ± 521.1	882.6 ± 535.5	0.33
Time to Insulin Peak (minutes)	53.9 ± 35.3	53.4 ± 33.1	48.6 ± 29.5	51.0 ± 30.0	0.74
C-Peptide AUC (nmol/L/120 min)	327.7 ± 105.1	342.7 ± 105.9	336.3 ± 109.5	338.7 ± 108.5	0.69
C-Peptide Peak (nmol/L)	3.4 ± 1.1	3.6 ± 1.1	3.5 ± 1.2	3.6 ± 1.2	0.31
Time to C-Peptide Peak (minutes)	76.8 ± 33.7	67.7 ± 30.9	66.0 ± 34.4	67.1 ± 31.9	0.31
Insulin Sensitivity Index	1.29 ± 1.17	1.30 ± 1.06	1.50 ± 1.24	1.43 ± 1.04	0.87

**p < 0.05, *p < 0.01 data presented as pairwise comparisons between each NNS preload and the seltzer water control

Rates of gastric emptying and intestinal glucose absorption were not different following Diet Rite Cola™ (Additional file 2: Figure S1) compared to seltzer water and subjective hunger and satiety ratings were similar between all test conditions (data not shown). Intestinal glucose absorption, rate of gastric emptying, and subjective hunger and satiety ratings were also similar following all four sucralose pre-treatment conditions (data not shown).

## Discussion

Our results demonstrate that a single exposure to a beverage containing sucralose, acesulfame-potassium, and other ingredients prior to an oral glucose load does not induce pronounced metabolic effects. However, GLP-1 secretion was enhanced after Diet Rite Cola™, and a statistically non-significant, but potentially biologically important increase of insulin was observed.

These results are consistent with our prior findings that Diet Rite Cola™ sweetened with sucralose and acesulfame-potassium enhanced GLP-1 secretion in healthy youth, as well as in young individuals with type 1 diabetes [26]. In the current study, we observed that neither sucralose alone nor the combination of sucralose with ace-K augmented the incretin response. Instead, only the ingestion of diet soda resulted in higher GLP-1 concentrations. This suggests that either the taste associated with diet soda or the effect of other ingredients are required to stimulate GLP-1 (Additional file 1: Table S1). We did not examine whether acesulfame-potassium in isolation elicits these metabolic responses. This sweetener differs from sucralose and aspartame in that it activates bitter taste receptors at lower concentrations [30]. Furthermore, the addition of aspartame in Diet Mountain Dew™ did not alter the observed results.

Animal studies have shown that NNS upregulate glucose transporters (GLUT2, SGLT1). However, we did not find a change in glucose uptake when measuring 3-OMG, which was added to the oral glucose load as a tracer. This may be due to﻿ the large 75 g glucose load administered, which may have ‘overloaded’ the system, as all glucose ingested is eventually absorbed. We also did not observe delayed gastric emptying, a known downstream effect of increased GLP-1 secretion. This is not entirely surprising, however, as the elevation of GLP-1 observed in our study (20 % increase) was considerably lower than what is typically observed following pharmacologic elevation of GLP-1 via DPP4 inhibitors (100 % increase) [31].

Despite the lack of statistical significance, it is noteworthy that all conditions with NNS given in combination resulted in marginally higher insulin AUCs (22-25 %). Pepino et al. [21] observed similar results with sucralose alone, and it is possible that her predominantly obese, female study participants may explain this difference. Interestingly, our participants in study arm 2 had higher baseline insulin secretion and may thus have been more susceptible to metabolic alterations following NNS exposure. Whether the insulin secretion observed in the current study and by Pepino et al. [21] was stimulated directly by sweet taste receptors on beta cells as shown in vitro [19] or via other mechanism remains entirely speculative. However, if our results are sustained with chronic NNS ingestion, then minor changes in insulin levels may contribute to eventual weight gain, as insulin is an anabolic hormone known to promote food intake and fat storage.

As alluded to above, considerable differences in study participants and methods may explain variable findings reported in the literature. Age, obesity, weight history and prior experience with sweet taste influence sweet taste perception [32–34] and hormonal responses. Dose, volume, composition, route of administration, and timing of NNS also vary between studies. Furthermore, dietary intake and physical activity may affect OGTT results, and are typically not standardized in acute studies. Polymorphisms in sweet taste receptor genes may also explain clinically relevant individual differences [35].

Our study was not designed to address the role of carbonation. We also did not standardize meals and physical activity prior to each visit and included individuals with a range of habitual NNS consumption. Finally, we did not adjust for multiple comparisons in the statistical analysis, and our study was not powered to detect changes in insulin. An inherent limitation of our study is also the inability to predict the consequences of prolonged NNS consumption.

The strengths of our study include testing NNS at varying doses, both in isolation and combined with other ingredients in diet soda. This allowed us to differentiate between potential effects due to the palatability and ingredient composition of diet soda and those due to NNS. The use of a crossover design allowed for control of intra-individual differences.

## Conclusions

The current study demonstrates that ingestion of diet sodas containing sucralose and acesulfame-potassium leads to subtle increases of glucose-stimulated GLP-1 release. While NNS only augmented GLP-1 in the context of diet soda, sucralose and acesulfame-potassium consistently augmented insulin levels even when administered in isolation (seltzer), although the effect was not statistically significant. Our findings reiterate the need for prospective, well-controlled, prolonged exposure trials to determine the role of diet soda and NNS in metabolic health and to differentiate between independent effects of NNS and those which may be due to other ingredients in diet soda and/or synergism between sweeteners and other ingredients.

## Additional files

Additional file 1: Table S1. Ingredients in diet sodas. (DOCX 11 kb)
Additional file 2: Figure S1. Serial data from OGTTs. Acetaminophen (A) and 3-O-methyl glucose (3-OMG) (B) are shown after ingestion of either Diet Rite Cola™ (*red square*), or seltzer water (*blue circle*) 10 min prior to a 75 g oral glucose load. (PDF 321 kb)

## Abbreviations

3-OMG: 3-*O*-methylglucose
AUC: Area under the curve
BMI: Body mass index
GC-MS: Gas chromatography–mass spectrometry
GIP: Gastric inhibitory peptide
GLP-1: Glucagon-like-peptide 1
NIDDK: National Institute for Diabetes and Digestive and Kidney Diseases
NIH: National Institutes of Health
NNS: Non-nutritive Sweeteners
OGTT: Oral glucose tolerance test
