# Supplementary material for: Hormonal responses to non-nutritive sweeteners in water and diet soda
Source: Nutr Metab (Lond). 2016 Oct 21;13:71. doi: 10.1186/s12986-016-0129-3 (PMC5073441; doi:10.1186/s12986-016-0129-3)
Supplement: Additional file 1: Table S1. — Ingredients in diet sodas. (DOCX 11 kb) [file 12986_2016_129_MOESM1_ESM.docx]

**Additional file 1: Table S1**. Ingredients in diet sodas

| **Ingredients** | **Diet Rite Cola™** | **Diet Mountain Dew™** |
| --- | --- | --- |
| Acacia Gum | X | X |
| Acesulfame-potassium | X | X |
| Aspartame |  | X |
| Brominated Vegetable Oil |  | X |
| Calcium Disodium EDTA |  | X |
| Carbonated Water | X | X |
| Caramel Color | X |  |
| Citric Acid | X | X |
| Citrus Pectin |  | X |
| Concentrated Orange Juice |  | X |
| Natural Flavors | X | X |
| Phosphoric Acid | X |  |
| Potassium Benzoate | X | X |
| Potassium Citrate | X | X |
| Sodium Citrate |  | X |
| Sucralose | X | X |
| Yellow #5 |  | X |
