# Supplementary material for: Hormonal responses to non-nutritive sweeteners in water and diet soda
Source: Nutr Metab (Lond). 2016 Oct 21;13:71. doi: 10.1186/s12986-016-0129-3 (PMC5073441; doi:10.1186/s12986-016-0129-3)
Supplement: Additional file 2: Figure S1. — Serial data from OGTTs. Acetaminophen (A) and 3-O-methyl glucose (3-OMG) (B) are shown after ingestion of either Diet Rite Cola™ (red square), or seltzer water (blue circle) 10 min prior to a 75 g oral glucose load. (PDF 321 kb) [file 12986_2016_129_MOESM2_ESM.pdf]

	Diet Rite Cola™	Seltzer Water	p-value
Acetaminophen AUC (µmol/L/120 min)	6031.3 ± 275.7	5681.4 ± 224.0	0.13
3-O-methyl glucose AUC (µg/mL/120 min)	10278.5 ± 435.09	10046.1 ± 357.18	0.52

A

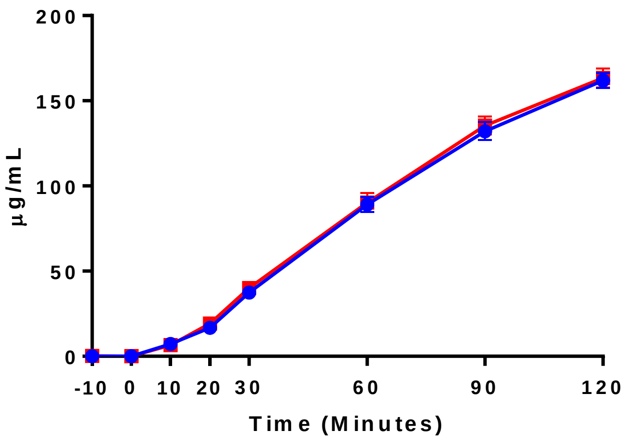

B

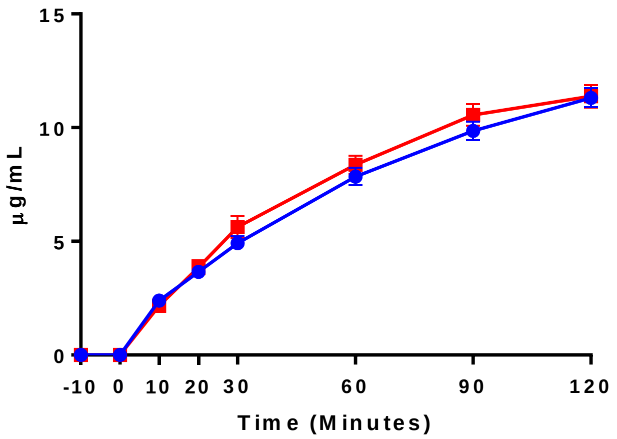

Supplemental Figure S1. Serial data from OGTTs. Acetaminophen (A) and 3-*O*-methyl glucose (3-OMG) (B) are shown after ingestion of either Diet Rite Cola™ (■), or seltzer water (●) 10 minutes prior to a 75 gram oral glucose load.
